# Macrophage polarization states in atherosclerosis

**DOI:** 10.3389/fimmu.2023.1185587

**Published:** 2023-05-03

**Authors:** Jiayong Wu, Shengping He, Zhengkun Song, Sikai Chen, Xuefeng Lin, Huimei Sun, Pengyu Zhou, Qinbao Peng, Songlin Du, Shaoyi Zheng, Xiu Liu

**Affiliations:** Department of Cardiovascular Surgery, Nanfang Hospital, Southern Medical University, Guangzhou, China

**Keywords:** atherosclerosis, macrophage polarization, biomolecules, inflammation, phenotype

## Abstract

Atherosclerosis, a chronic inflammatory condition primarily affecting large and medium arteries, is the main cause of cardiovascular diseases. Macrophages are key mediators of inflammatory responses. They are involved in all stages of atherosclerosis development and progression, from plaque formation to transition into vulnerable plaques, and are considered important therapeutic targets. Increasing evidence suggests that the modulation of macrophage polarization can effectively control the progression of atherosclerosis. Herein, we explore the role of macrophage polarization in the progression of atherosclerosis and summarize emerging therapies for the regulation of macrophage polarization. Thus, the aim is to inspire new avenues of research in disease mechanisms and clinical prevention and treatment of atherosclerosis.

## 1 Introduction

Cardiovascular disease (CVD) is the leading cause of mortality and morbidity worldwide (1). Atherosclerosis is the primary pathological basis of CVD (2); therefore, it is critical to thoroughly investigate the pathogenesis of atherosclerosis. The development of atherosclerosis involves many cells such as endothelial cells, vascular smooth muscle cells, and macrophages. Macrophages in particular are involved in the entire pathological process of atherosclerosis, playing a central role in progression, from the formation of foam cells in the early stage to the rupture of atherosclerotic plaques in the late stage (3). Studies have shown that the effect of macrophages on atherosclerotic plaques is not only determined by the number of infiltrated macrophages but also by their polarization state and the relative proportion of different phenotypes (4, 5). The progression of atherosclerosis is not completely delayed by lipid-lowering therapies, and regulating macrophage polarization and phenotype proportions in plaques is hypothesized to be a promising therapeutic opportunity. Herein, we summarize the role of macrophage polarization in the development of atherosclerosis and advances in therapeutic regulation of macrophage polarization.

## 2 Progression of atherosclerosis

**Figure 1:**
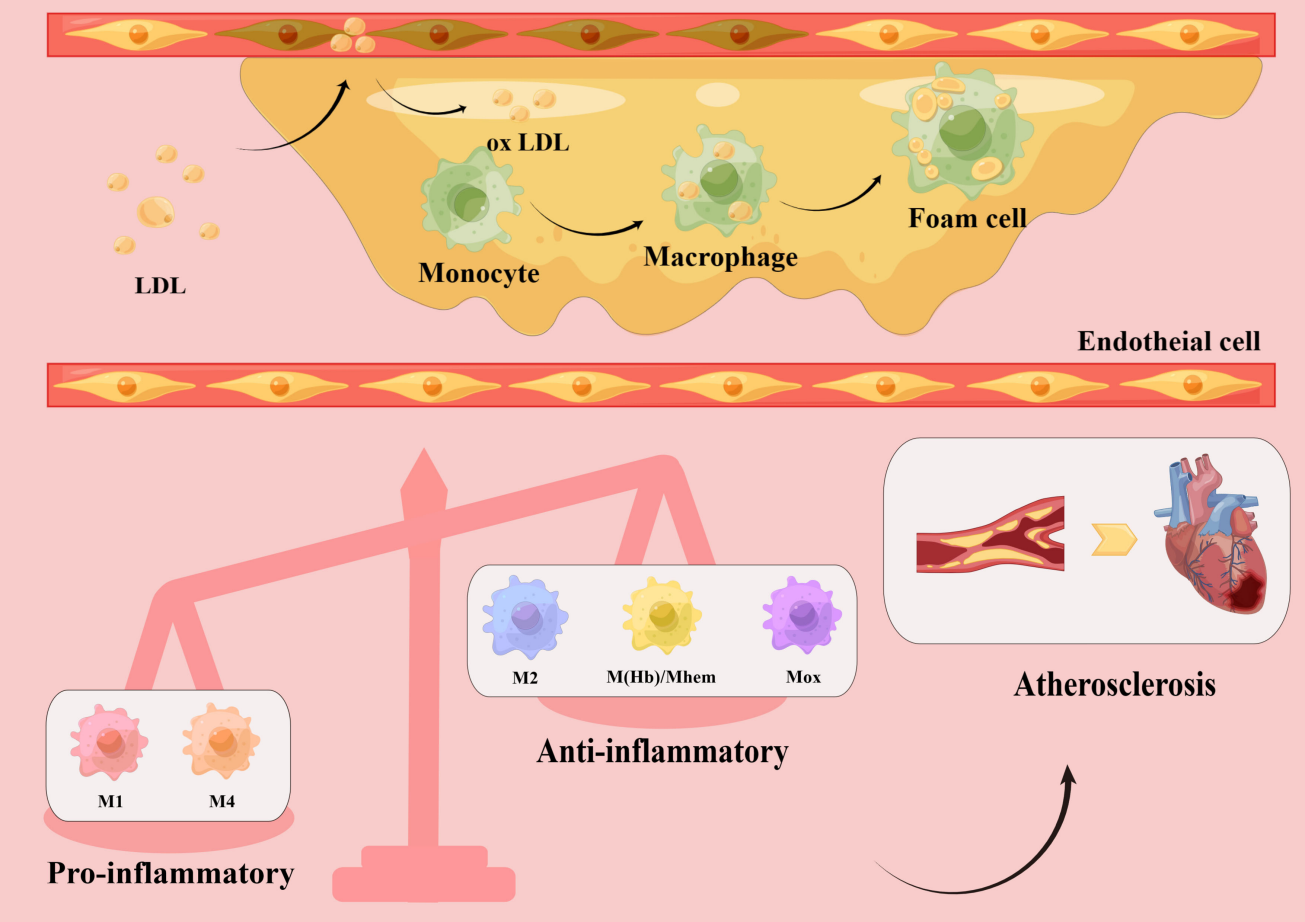
Macrophage polarization in atherosclerotic plaques. Low-density lipoprotein (LDL) enters the vascular intima through damaged endothelial cells and is modified into oxidized LDL, which activates endothelial cells to express chemokines and adhesion molecules, recruiting circulating monocytes. Subsequently, monocytes differentiate into macrophages in a local microenvironment rich in growth factors and pro-inflammatory cytokines. Macrophages rapidly recognize and engulf oxidized LDL, transforming into foam cells and accumulating into plaques. Different subsets of macrophages, including pro-inflammatory macrophages (M1, M4) and anti-inflammatory macrophages (M2, M(Hb), Mhem, Mox), have been found in atherosclerotic plaques. The increased number of pro-inflammatory macrophages and decreased number of anti-inflammatory macrophages lead to a series of inflammatory responses and promote atherosclerotic plaque formation, which eventually lead to cardiovascular disease.

Atherosclerosis is a chronic inflammatory disease that primarily affects large and medium arteries (6–9). Under the influence of major risk factors, damaged endothelial cells allow low-density lipoprotein cholesterol (LDL-C) to enter the intima, where it is modified into oxidized LDL-C. Oxidized LDL-C activates the expression of chemokines and adhesion factors in endothelial cells, recruiting circulating monocytes which, in a local microenvironment rich in growth factors and proinflammatory cytokines, subsequently differentiate into macrophages. Through scavenger receptors on their surface, macrophages quickly identify and engulf oxidized LDL-C, transforming into foam cells that establish the earliest atherosclerotic lesions (10). As the disease progresses, the accumulation of foam cells, local necrosis, and the formation of a fibrous cap lead to the development of stable or unstable plaques (11). In the late stage of atherosclerosis, owing to hemodynamic changes, stress, and inflammatory responses, plaque instability eventually causes plaque rupture, bleeding, and thrombosis. Thus, the formation and accumulation of foam cells are key steps in the development of atherosclerosis (12). As a result, most therapeutic trials have focused on decreasing lipid levels to reduce the number of foam cells (13). However, it is becoming apparent that simply reducing lipids does not completely prevent the progression of atherosclerosis, and macrophages have become a new potential therapeutic target (**Figure 1**).

## 3 Macrophage polarization in atherosclerotic plaques

### 3.1 Macrophage polarization

Macrophages are one of the major immune cells in the human body (14). They participate in the physiological and pathological processes of immunity and inflammatory repair by regulating inflammation and clearing infection through antigen presentation, polarization, and phagocytosis. Macrophage polarization refers to the process of changing phenotype and demonstrating differential functions by adapting to the surrounding environment. In the complex microenvironment of atherosclerotic plaques, macrophages are simultaneously stimulated by a variety of signals and accordingly polarize into different subtypes (15), which play different roles in the progression of atherosclerosis (16). The macrophage subtypes currently identified include M1, M2, M (Hb), Mhem, Mox, and M4.

M1 macrophages are activated by multiple factors, including interferon-γ (IFN-γ) and lipopolysaccharide (LPS) (17, 18), and produce and secrete pro-inflammatory cytokines, such as tumor necrosis factor (TNF)-α, interleukin (IL)-1β, and IL-6, as well as nitric oxide (NO) and reactive oxygen species (ROS). Functionally, M1 macrophages participate in the clearance of pathogens and induce tissue damage by activating the nicotinamide adenine dinucleotide phosphate (NADPH) oxidase complex, leading to ROS production (19). Simultaneously, M1 macrophages express chemokine receptor ligands, such as CXC chemokine ligand (CXCL)-9, CXCL-10, and CXCL-5, to promote the recruitment of Th1 and natural killer cells, resulting in a sustained inflammatory response critical for eliminating cellular pathogens (20). However, in the aseptic environment of atherosclerosis, proinflammatory M1 macrophages cause sustained inflammation, damaging surrounding tissues (21).

In contrast, the main function of M2 macrophages is to inhibit inflammation, clear cell debris and apoptotic cells, and contribute to tissue repair and fibrosis (22–24). Activated by IL-4 and IL-13, they secrete anti-inflammatory factors, such as IL-10 and TNF-β, and express chemokines, such as C-C Motif Chemokine Ligand (CCL) 17, CCL22, and CCL24, thus initiating a functional anti-inflammatory regulatory mechanism to counteract the chronic inflammatory response caused by M1 macrophages (25, 26).

M2 macrophages can be divided into four subtypes according to differing *in vitro* stimulation factors: M2a, M2b, M2c, and M2d. The M2a subtype can be induced by IL-4 and IL-13 and expresses high levels of glucocorticoid receptors. This subtype also secretes pro-fibrotic factors such as fibronectin, insulin-like growth factor, and transforming growth factor-β (TGF-β) (27, 28), thus promoting tissue repair. Consequently, they are usually referred to as wound healing macrophages (29). The M2b subtype can be induced by immune complexes, Toll-like receptor (TLR) agonists, or IL-1 receptor ligands. This subtype can produce both anti-inflammatory factors, such as IL-10 and IL-12, and pro-inflammatory factors, such as IL-1β, IL-6 and TNF-α (30, 31). M2c macrophages are induced by glucocorticoids and IL-10 and have strong anti-inflammatory activity against apoptotic cells by releasing large amounts of IL-10 and TGF-β (7). Since M2b and M2c macrophages both show high expression of Mer receptor tyrosine kinase and present efficient phagocytic capabilities, they are also referred to as regulatory macrophages (31). The fourth subtype, M2d macrophages, can be induced by TLR agonists via adenosine A2A receptor stimulation. This type of macrophage inhibits the production of pro-inflammatory cytokines and induces the secretion of anti-inflammatory cytokines such as IL-10 and IL-12 (32).

M (Hb) and Mhem macrophages differ from lipid-core macrophages and coexist in the bleeding sites of newly formed vessels or unstable plaques (33). M(Hb) macrophages, produced by hemoglobin stimulation, typically express high levels of mannose and CD163 receptors, and participate in the clearance of hemoglobin/haptoglobin (Hb/Hp) complex after plaque hemorrhage (34). After endocytosis of the Hb/Hp complex and erythrocytes, the released heme can prime macrophages toward a Mhem macrophage phenotype (35). In intraplaque hemorrhages, they engulf and recycle erythrocyte remnants and hemoglobin, may be induced by hemoglobin, albumin, and CD163, and subsequently induce a liver X receptor (LXR)-α/adenosine triphosphate binding cassette (ABC) transporter ABCA1/apolipoprotein E (APOE) cascade by activating transcription factor 1 (35). The high expression of LXRα, LXRβ, and ABC transporters ABCA1 and ABCG1, which are responsible for cholesterol efflux, indicates that this subtype can prevent foam cell formation and provide certain protective effects against atherosclerosis (34, 35).

Similarly, Mox macrophages are induced by oxidized phospholipids, characterized by reduced phagocytic activity and chemotaxis, and the expression of antioxidant enzymes such as heme oxygenase-1, thioredoxin reductase-1, and sulfiredoxin-1 are significantly upregulated by nuclear factor erythroid 2-related factor 2. This suggests that Mox macrophages may also be anti-atherosclerotic and resistant to oxidative stress (36).

M4 macrophages are induced by CXCL-4 and primarily express CD68, calcium-binding protein S100A8, and matrix metalloproteinase (MMP) 7 in the arterial adventitia and intima. They are accompanied by the expression of pro-inflammatory factors such as MMP-12, IL-6, and TNF-α (37). In addition, CXCL-4 induces inflammation and aggravates atherosclerosis by suppressing CD163 (38), indicating the pro-atherogenic role of this subtype (37, 39).

In summary, it is evident that macrophages do not always promote atherosclerosis, and that different subtypes participate in the development of atherosclerosis through different pathways. Notably, polarized macrophages possess plasticity and can switch their phenotypes and functions according to the microenvironment. They can also modulate the phenotypes of other macrophage subsets, thus collectively inducing plaque progression or regression (40, 41) (**Figure 1**).

### 3.2 M1/M2 macrophages polarization in atherosclerosis

Although macrophages can polarize into many specific phenotypes, most studies still use the M1/M2 classification to summarize their characteristics. Study shows that macrophages can polarize into two distinct functional subpopulations in response to atherosclerotic inflammatory responses: pro-inflammatory M1 and anti-inflammatory M2 (42). In the early stages of the lesion, M2 macrophages are predominantly found in the plaque, which tend to be stable. Studies have shown that M2 macrophages promote plaque stability by secreting collagen and enhancing the clearance of apoptotic cells. However, as the lesions progress, the number of M2 macrophages decrease (43), the number of M1 macrophages gradually increase, the secretion of pro-inflammatory factors increase, the plaque is prone to rupture (40), and the expression of cholesterol transporter-related protein ABCA1 decreases, leading to the obstruction of cholesterol efflux. The resulting accumulation of cholesterol further promotes macrophage activation and M1 polarization, causing a vicious cycle. In addition, M1 macrophages can secrete MMPs, such as MMP2 and MMP9, leading to the degradation of extracellular matrix in the plaque and causing plaque instability and rupture, which can increase the incidence of acute cardiovascular events (44).

The distribution of M1 and M2 subtypes in different regions of the atherosclerotic plaque also varied; for example, M1 macrophages are more likely to infiltrate the shoulder of the plaque, which is prone to rupture (45). In the fibrous cap region, the proportions of the two subtypes are similar; the protective and destructive effects are offset, thus maintaining plaque stability (45, 46). In late lesions, M1 macrophages are predominantly found around the necrotic core of the plaque, while M2 macrophages are more prevalent near newly formed blood vessels (40, 47). More importantly, the M1/M2 ratio changed dynamically during the development of atherosclerosis, and the subtypes can be transformed into each other (40, 41). Khallou-Laschet et al. conducted *ex vivo* re-polarization experiments and found that fully polarized macrophages could be re-polarized into another subtype; that is, M1 could be polarized into M2 after IL-4 induction, and M2 could be polarized into M1 after LPS and IFN-γ induction (40). This experiment reveals that polarized M1 and M2 macrophages retain their original plasticity, suggesting that the differentiation of macrophages from M1 to M2 may promote the stabilization and regression of atherosclerotic plaques (48, 49). Recent study has shown that upregulation of NFATc3 converts proinflammatory macrophages into those with an anti-inflammatory phenotype, consequently inhibiting foam cell formation and preventing atherosclerosis (4). Kruppel-like factor (KLF) 2 is also reported to be involved in the transformation of M2 macrophages into pro-inflammatory M1 macrophages (50). Similarly, hepatocyte growth factor (HGF) is capable of tissue repair by altering M1 macrophages into M2 macrophages, but the molecular mechanism is still unclear (51).

**Figure 2:**
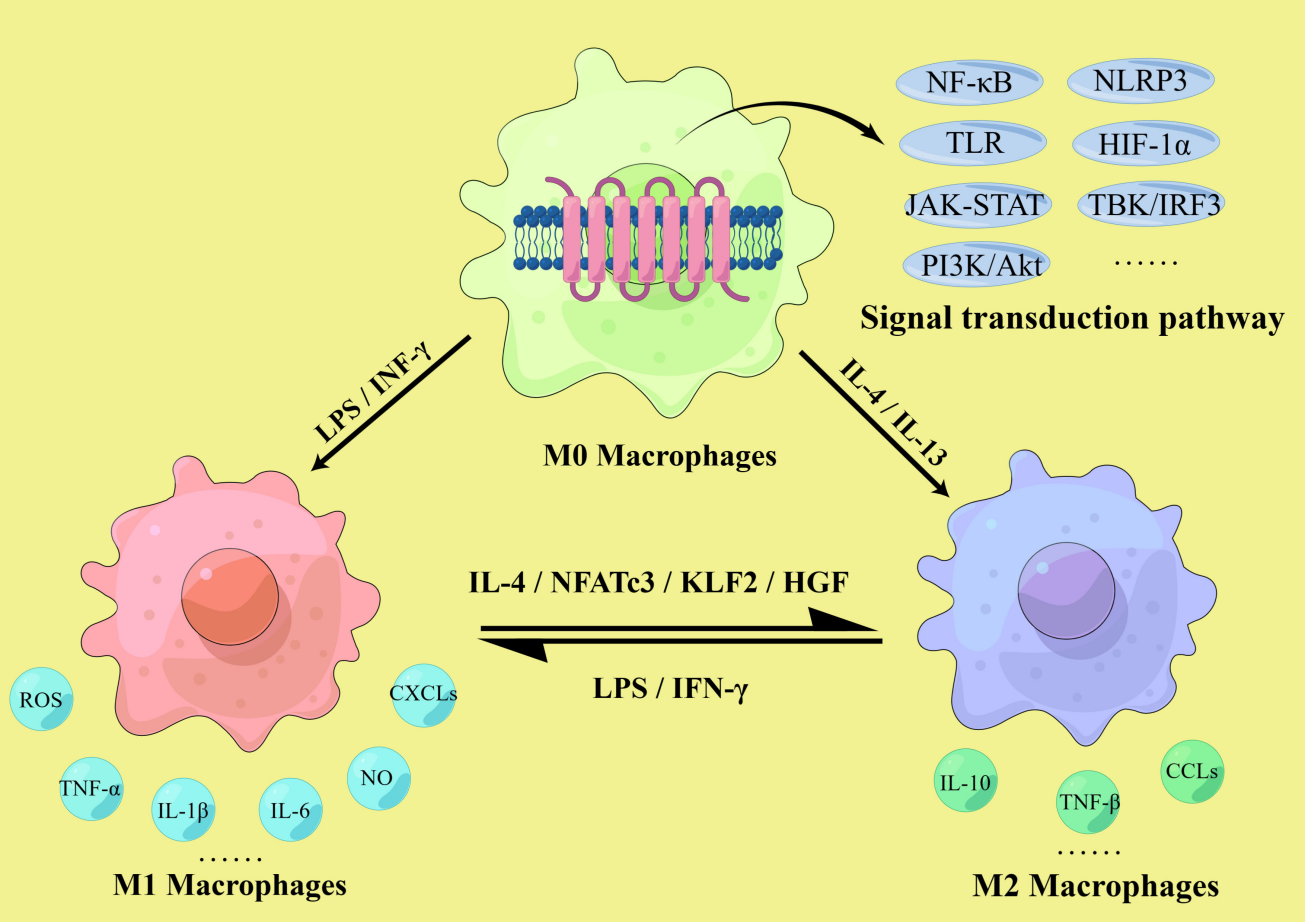
M1/M2 macrophages polarization. The polarization of macrophages in atherosclerosis involves multiple signaling pathways, including the NLRP3 inflammasome, Toll-like receptors (TLR) and hypoxia inducible factor-1 α (HIF-1α) mediated pathway, NF-κB signaling pathway, the JAK-STAT pathway, TBK/IRF3 pathway and PI3K-Akt/PKB pathway. M1 macrophages are activated by IFN-γ and LPS and secrete pro-inflammatory cytokines such as TNF-α, IL-1β, and IL-6, as well as NO, ROS, and various chemokine receptor ligands. M2 macrophages are activated by IL-4 and IL-13, secrete anti-inflammatory cytokines such as IL-10 and TNF-β, and express relevant chemokines to initiate anti-inflammatory mechanisms. The ratio of M1/M2 is dynamic, and they can transform into each other. M1 could be polarized into M2 after IL-4, KLF2 and hepatocyte growth factor (HGF) induction, and M2 could be polarized into M1 after LPS and IFN-γ induction.

These findings suggest that macrophage polarization is closely related to atherosclerosis in both temporal and spatial dimensions. While M1 macrophages are mainly pro-inflammatory and accelerate atherosclerotic development, M2 macrophages are mainly anti-inflammatory, promote tissue repair, and can inhibit disease progression to some extent. Thus, the imbalance of the M1/M2 ratio in the lesion affects the development of atherosclerosis, and regulating the direction of macrophage polarization has significant implications in the prevention and treatment of atherosclerosis (**Figure 2**).

## 4 Macrophage polarization as a target for treating atherosclerosis

Many factors are associated with the development, progression and outcome of atherosclerosis, such as cell apoptosis, ferroptosis, metabolic reprogramming, epigenetic modifications, miRNA, autophagy and pyroptosis, all of which can affect macrophage polarization (4, 32, 52). Although different subtypes of macrophages may show different sensitivity to these factors, polarization plays an irreplaceable role in plaque formation and is crucial for the prevention and treatment of atherosclerosis. Normally, M1 and M2 macrophages are in dynamic equilibrium, maintaining the M1/M2 ratio within a certain range to ensure the effective function of related immune responses. However, under the long-term stimulation of the plaque microenvironment, this balance is gradually disturbed. In atherosclerosis, many factors affect macrophage polarization, including cytokines, enzymes, transcription factors, related proteins and receptors such as interleukins, tumor necrosis factors, interferons, Kruppel-like factors, immunoglobulins and chemokine receptor ligands (53–55), as well as various signaling pathways, including the NOD-like receptor family pyrin domain-containing 3 (NLRP3) inflammasome, the TLR and hypoxia inducible factor-1 α (HIF-1α) mediated pathway, nuclear factor (NF)-κB signaling (56–58), the granulocyte-macrophage colony-stimulating factor-related pathway, the JAK-STAT pathway, TBK/IRF3 pathway and the phosphatidylinositol 3-kinase (PI3K)-protein kinase B (Akt/PKB) pathway (59–61). In addition, the lack of the Akt subtype Akt2 is beneficial for suppressing M1 polarization and promoting M2 polarization, leading to a decrease in the M1/M2 ratio, thereby delaying the progression of atherosclerosis (56). Based on these conditions, it is theoretically possible to regulate macrophage polarization to prevent and treat atherosclerosis (**Figure 2**).

### 4.1 Biomolecules

New potential therapeutic targets for treating atherosclerosis including regulating the phenotype and relative proportion of macrophages in plaques is needed. Kallistatin has been recognized as a plasma protein with anti-inflammatory functions, it can significantly stimulate the expression and differentiation of M2 markers, and reduce the expression of M1 markers, thereby inhibiting the formation of atherosclerotic plaques (62). C3a anaphylatoxin receptor (C3aR) signaling has protective roles in various inflammatory-related diseases. Wei et al. found that C3aR also has a protective effect in the development of atherosclerosis through experiments, and revealed that C3aR confers the protection through C3a/C3aR axis-mediated negative regulation of proinflammatory responses and modulation of macrophage toward the anti-inflammatory phenotype (63). KLF4 is an important transcription factor involved in cell growth, differentiation, and proliferation and has been shown to play a significant role in regulating cardiovascular diseases. Li et al. found that KLF4 inhibition and increased expression of cholesterol-25-hydroxylase (Ch25h) and LXR, followed by activation of the KLF4-Ch25h/LXR pathway, can increase the reverse transport of cholesterol in blood vessels, thereby inducing macrophage polarization towards the M2 phenotype and suppressing vascular inflammatory responses (64). Likewise, IL-19 can activate key pathways of M2 macrophage polarization, including those of signal transduction and transcription activator (STAT) 3, STAT6, and peroxisome proliferator-activated receptor (PPAR)-γ, and can reduce cytokine-induced systemic inflammation to prevent the progression of atherosclerotic plaques (65). Similarly, Oleoylethanolamide is an endogenous agonist of PPAR-α and, under inflammatory conditions, can promote M2 and inhibit M1 macrophage polarization, and stabilize plaques by activating the adenosine 5’-monophosphate (AMP)-activated protein kinase (AMPK)-PPARα signaling pathway (66). IgE may induce M1 macrophage polarization by binding to the high-affinity IgE receptor and sodium hydrogen exchanger 1 receptor. Consequently, IgE deficiency can reduce atherosclerotic inflammation and promote M2 macrophage polarization; therefore, targeting IgE therapy for atherosclerosis is an attractive therapeutic route (67). Cao et al. demonstrated that, in macrophages, the absence of histone deacetylase 9 can promote M2 polarization and downregulate M1-associated inflammatory gene expression by upregulating PPAR-γ expression via chromatin remodeling (68). Palmitoylethanolamide is an endogenous fatty acid mediator with anti-inflammatory properties that has been shown to reduce M1 macrophage polarization and enhance the stability of murine atherosclerotic plaques (69). Galectin-12 has been shown to induce M2 macrophage polarization, suppressing foam cell formation and pro-inflammatory cytokine production, thereby reducing the formation of atherosclerotic plaques. However, the precise signaling pathways by which galectin-12 mediates M2 macrophage polarization remain to be elucidated (70). Lastly, microRNAs such as microRNA-494-3p, MicroRNA-216a, and miR-27b, are expressed in atherosclerotic plaques and regulate macrophage polarization by negatively regulating gene expression through inhibition of the translation process or enhanced mRNA degradation, which not only reduces the formation of atherosclerotic plaques but also increases plaque stability (71–73). Although these biomolecules are currently not available for clinical use, they provide a new perspective on reducing the plaque area and enhancing plaque stability by interfering with macrophage polarization.

### 4.2 Therapeutic agents

In addition to the above biomolecules, recent studies have found that many drugs, including conventional Western and traditional Chinese medicines, play a role in regulating macrophage polarization. Further exploration into the mechanisms of action of these medicines may provide promising new targets for the prevention and treatment of atherosclerosis.

Galectins are a group of animal lectins that can specifically bind to β-galactoside. They play an important role in many physiological and pathological processes, such as cell adhesion, cell apoptosis, inflammatory response, and tumor metastasis. Recent studies have found that Galectin-3 has a prominent protective effect in regulating macrophage polarization and invasive ability, thus delaying plaque progression (74). In addition, prolonged and frequent treatment with anti-Gal-2 nanobodies reduced plaque size, slowed plaque progression, and modified the phenotype of plaque macrophages toward an anti-inflammatory profile (75). The anti-atherosclerotic and anti-inflammatory effects of AVE0991, a non-peptide angiotensin 1-7 mimetics, maybe attributed to its suppression of pro-inflammatory M1 macrophage differentiation (76). VX765, a well-established inhibitor of caspase 1, which robustly restrains caspase 1-mediated interleukin-1β production. Importantly, VX765 potentiates IL4-induced M2 polarization and increases expression of mannose receptor 1 and arginase 1, two well-recognized markers of M2 macrophages (77). Statins, such as rosuvastatin, are cholesterol-lowering drugs commonly used in clinical practice. Rosuvastatin can increase autophagy by inhibiting the PI3K/Akt/mammalian target of rapamycin signaling pathway, leading to increased expression of cholesterol efflux transporters ABCA1 and ABCG1, reduced foam cell formation, increased expression of M2 macrophage markers arginase-1 and CD206, and decreased expression of inducible nitric oxide synthase (iNOS) in the vascular wall, thus inducing macrophage polarization towards the anti-inflammatory M2 phenotype (78). Rivaroxaban, a novel oral anticoagulant, has been shown to inhibit M1 macrophage polarization by inhibiting the protease activated receptor 2 (PAR2)/Akt/HIF-1α signaling pathway induced by FXa (factor Xa) (79). Sitagliptin, a widely used drug for the treatment of type 2 diabetes, has been shown to promote macrophage polarization towards the M2 phenotype via chemokine stromal cell-derived factor-1/C-X-C chemokine receptor type 1 (SDF-2/CXCR1) signaling, thereby attenuating early atherosclerotic lesion formation (80). Similarly, a new class of anti-diabetic drugs, sodium-glucose co-transporter 2 (SGLT2) inhibitors, such as dapagliflozin, have been shown to inhibit the overexpression of TLR-4 induced by LPS and the activation of NF-κB in human endothelial cells and differentiated macrophages, thereby promoting the conversion of M1 macrophages to the M2 phenotype (81). Prostaglandin E2 has been found to promote M2 macrophage polarization by activating the cyclic AMP response element binding protein/brain-derived neurotrophic factor/tyrosine kinase receptor B (CREB-1) signaling pathway, thereby alleviating diabetic atherosclerosis (82). Melatonin, an indolamine hormone, has been shown to inhibit M1 and promote M2 polarization by differential regulation of the AMPKα/STAT pathway in a RORα (Retinoid acid receptor-related orphan receptor-α)-dependent manner (83). Protocatechuic acid (PCA) is a flavonol that possesses significant protection against atherosclerosis. PCA inhibits M1 polarization by suppressing PI3K/Akt-mediated NF-κB activation and induces STAT6 phosphorylation and PPARγ activation, resulting in enhanced M2 activation (84). Inosine, a cellular metabolism-enhancing agent, has been proposed for the treatment of atherosclerosis owing to its ability to inhibit the p38 mitogen-activated protein kinase (MAPK)/NF-κB signaling pathway (85). However, the influence of inosine on macrophage polarization remains to be explored.

Artesunate, a sesquiterpene lactone endoperoxide isolated from Chinese herbal medicine, displays excellent anti-inflammatory activity, which can inhibit M1-like macrophage polarization via regulating HIF-1α and NF-κB signaling pathways (86). Ginsenoside Rb1, an active ingredient in ginseng, was found by Zhang et al. to increase the expression of M2 macrophage markers arginase-1 and CD206 and to inhibit the expression of M1-related iNOS *in vitro* (87). *In vivo* experiments in an ApoE^-/-^ murine model of atherosclerosis showed that Rb1 intervention may enhance the stability of atherosclerotic plaques by promoting M2 polarization through increased secretion of IL-4 and IL-13 and phosphorylation of STAT6 (87). Curcumin, an active ingredient in turmeric rhizomes, can convert the pro-inflammatory M1 subtype to the anti-inflammatory M2 subtype via the TLR4/MAPK/NF-κB signaling pathway. Curcumin also affects human myeloid leukemia mononuclear cells (THP-1) by reducing the number of M1 macrophages induced by LPS and promoting the transformation of macrophages into the M2 subtype (88, 89). Crocin, a constituent of saffron, not only exerts anti-atherosclerotic effects by reducing oxidized LDL but also induces M2 macrophage polarization in a rat model of vitamin D3-induced coronary atherosclerosis (90). Furthermore, polydatin, a bioactive ingredient extracted from the roots of the Reynoutria japonica Houtt, can inhibit multiple signaling pathways including the NF-κB and TLR pathways, reduce the release of pro-inflammatory factors, decrease lipid deposition and foam cell formation, and exert anti-oxidative stress effects, thereby playing a multifaceted anti-atherosclerotic role (91, 92). Numerous studies have found that traditional Chinese medicine, both the active monomeric components and mixed compounds, can affect macrophage polarization through various signaling pathways. However, specific mechanisms of action for some of these remain to be elucidated.

## 5 Summary and future directions

The occurrence and development of atherosclerosis is a very complex process. As lipid-lowering therapy cannot completely delay the progression of atherosclerosis, and macrophage polarization is involved in various stage of atherosclerosis. Targeting macrophage polarization may be an effective method for the prevention and treatment of atherosclerosis, which is beneficial to the outcome of atherosclerotic cardiovascular disease. Multiple studies have shown that macrophage subtype imbalances in atherosclerosis are closely related to disease progression. Therefore, regulating macrophage polarization and promoting phenotypic transformation to anti-inflammatory subtypes, and inhibiting macrophage death through key molecules such as apoptosis, necrosis, iron death, autophagy, and pyroptosis, are expected to be effective approaches for the prevention and treatment of atherosclerosis. Moreover, the recently developed single-cell analysis technology can be used to further elucidate macrophage phenotypes and functions, and analyze the specific role of macrophages in plaques. However, as there are many differences in the diversity of macrophage phenotypes and functions, as well as the occurrence and development of atherosclerotic plaques between mice and humans, further *in vivo* and clinical studies are needed to elucidate how modulation of reducing M1/M2 macrophage ratio may be an effective approach for atherosclerosis treatment. Therefore, targeted treatment of macrophages as a strategy to prevent plaque progression and even accelerate plaque regression while reducing side effects is a compelling avenue for future research.

## Author contributions

JW, SH, SZ and XL contributed to conception and design of the study. JW and ZS organized the database. JW and XL wrote the first draft of the manuscript. SC, XFL, HS, QP, SD, PZ and XL wrote sections of the manuscript. SH and SZ created the figure and table. All authors contributed to the article and approved the submitted version.

## Glossary

**Table:**

CVD	cardiovascular disease
LDL-C	low-density lipoprotein cholesterol
ox LDL-C	oxidized low-density lipoprotein cholesterol
IFN-γ	Interferon - gamma
LPS	lipopolysaccharide
TNF-α	tumor necrosis factor-alpha
IL-1β	interleukin-1 beta
IL-6	interleukin-6
NO	nitric oxide
ROS	reactive oxygen species
NADPH	nicotinamide adenine dinucleotide phosphate
CXCL-5	CXC chemokine ligand-5
CXCL-9	CXC chemokine ligand-9
CXCL-10	CXC chemokine ligand-10
IL-4	interleukin-4
IL-10	interleukin-10
IL-13	interleukin-13
TNF-β	tumor necrosis factor-beta
CCL17	C-C Motif Chemokine Ligand 17
CCL22	C-C Motif Chemokine Ligand 22
CCL24	C-C Motif Chemokine Ligand 24
IGF	insulin-like growth factor
TGF-β	transforming growth factor-beta
ICs	immune complexes
TLR	Toll-like Receptor
MerTK	Mer receptor tyrosine kinase
IPH	intraplaque hemorrhage
LXR	liver X receptor
ABC	Adenosine Triphosphate binding cassette
HO-1	heme oxygenase-1
TrxR1	thioredoxin reductase-1
Srxn1	sulfiredoxin-1
Nrf2	nuclear factor erythroid 2-related factor 2
CXCL-4	CXC chemokine ligand-4
MMP-7	metalloproteinase 7
MMP-12	metalloproteinase 12
NF-κB	nuclear factor kappa-B
GM-CSF	granulocyte-macrophage colony-stimulating factor
PI3K	phosphatidylinositol 3-kinase
PKB	protein kinase B
KLF-4	kruppel-like factor 4
Ch25h	cholesterol-25-hydroxylase
STAT3	signal transduction and transcription activator 3
STAT6	signal transduction and transcription activator 6
PPAR-γ	peroxisome proliferator-activated receptor gamma
OEA	Oleoylethanolamide
PPAR-α	peroxisome proliferator-activated receptor alpha
NHE1	sodium hydrogen exchangers 1
FcϵR1	high affinity IgE receptor
HDAC9	histone deacetylase 9
PEA	Palmitoylethanolamide
mTOR	mammalian target of rapamycin
Arg1	arginase-1
HIF1α	hypoxia inducible factor-1 alpha
SGLT-2	sodium-glucose co-transporter 2
DAPA	dapagliflozin
PGE2	Prostaglandin E2
CREB	cyclic AMP response element binding protein
BDNF	brain-derived neurotrophic factor
TrkB	tyrosine kinase receptor B
PCA	Protocatechuic Acid
MAPK	mitogen-activated protein kinase
INOS	inducible nitric oxide synthase
VD3	vitamin D3
